# Supplementary material for: Mutant ASXL1 cooperates with BAP1 to promote myeloid leukaemogenesis
Source: Nat Commun. 2018 Jul 16;9:2733. doi: 10.1038/s41467-018-05085-9 (PMC6048047; doi:10.1038/s41467-018-05085-9)
Supplement: Supplementary file 1 — Supplementary Information [file 41467_2018_5085_MOESM1_ESM.pdf]

# Supplementary Information

**Mutant ASXL1 cooperates with BAP1 to promote myeloid leukaemogenesis**

Asada et al.

Supplementary Figure 1

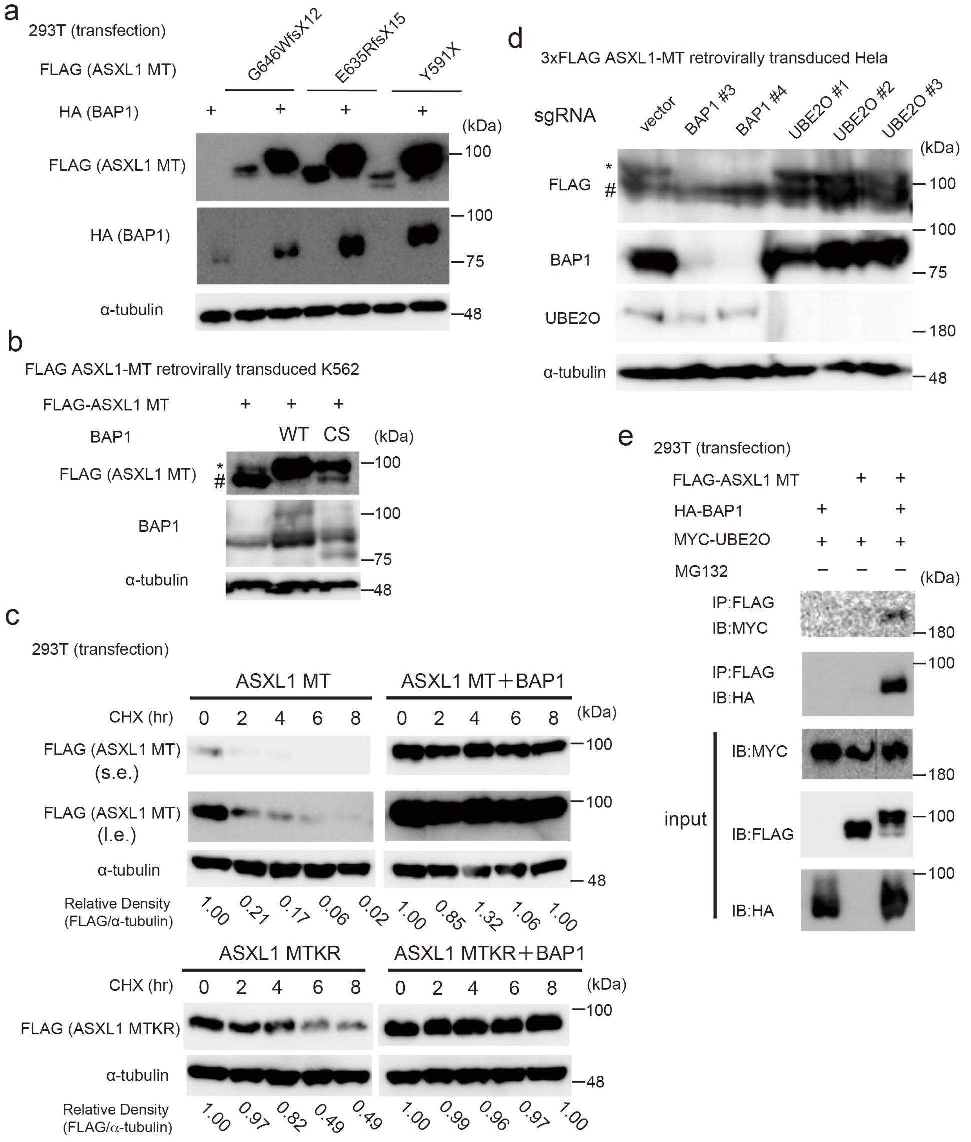


**Supplementary Figure 1. BAP1 induces monoubiquitination of ASXL1-MT. (a)** 293T cells were transfected with mutant ASXL1 forms (G646WfsX12, E635RfsX15 and Y591X) together with vector or HA-BAP1. 48hr after transfection, cells were harvested and cell lysates extracted from them were subjected to immunoblotting with anti-FLAG, anti-HA, and anti-tubulin antibodies. BAP1 overexpression caused the mobility shift of all these ASXL1 mutants. **(b)** K562 cells were retrovirally transduced with FLAG-ASXL1-MT in combination with vector, wild-type BAP1 (WT) or BAP1-C91S (CS), and cell lysates from them were subjected to immunoblotting with anti-FLAG and anti-BAP1 antibodies. Expression of BAP1 and BAP1-C91S promoted the mobility shift of ASXL1-MT, displaying predominantly the upper band (*). **(c)** 293T cells were transfected with FLAG-ASXL1-MT or FLAG-ASXL1-MTKR together with vector or HA-BAP1. 48hr later, cells were treated with 50 μg/ml cycloheximide (CHX) for the indicated times, and cell extracts were analyzed with anti-FLAG and anti-alpha-tubulin antibodies. The band intensities of FLAG relative to alpha-tubulin are shown. The value of FLAG/alpha-tubulin without CHX treatment was set to 1. **(d)** Hela cells were retrovirally transduced with 3xFLAG-ASXL1-MT (coexpressing GFP). $GFP^{+}$ cells were sorted by FACS Aria and were then transduced with control vector, two independent sgRNAs targeting human BAP1, or three independent sgRNAs targeting human UBE2O. Cell lysates extracted from them were subjected to immunoblotting with anti-FLAG, anti-BAP1, anti-UBE2O and anti-tubulin antibodies. BAP1 depletion led to disappearance of monoubiquitinated ASXL1-MT (*), leaving only the non-ubiquitinated form (#) **(e)** 293T cells were transfected with Myc-UBE2O together with vector or FLAG-ASXL1 MT and vector or HA-BAP1. Total cell lysates were immunoprecipitated with anti-FLAG M2 antibody, and UBE2O bound to ASXL1 was detected by anti-Myc. In the absence of proteasome inhibitor MG132, the interaction between ASXL1-MT and UBE2O was detected only with BAP1 overexpression.

Supplementary Figure 2

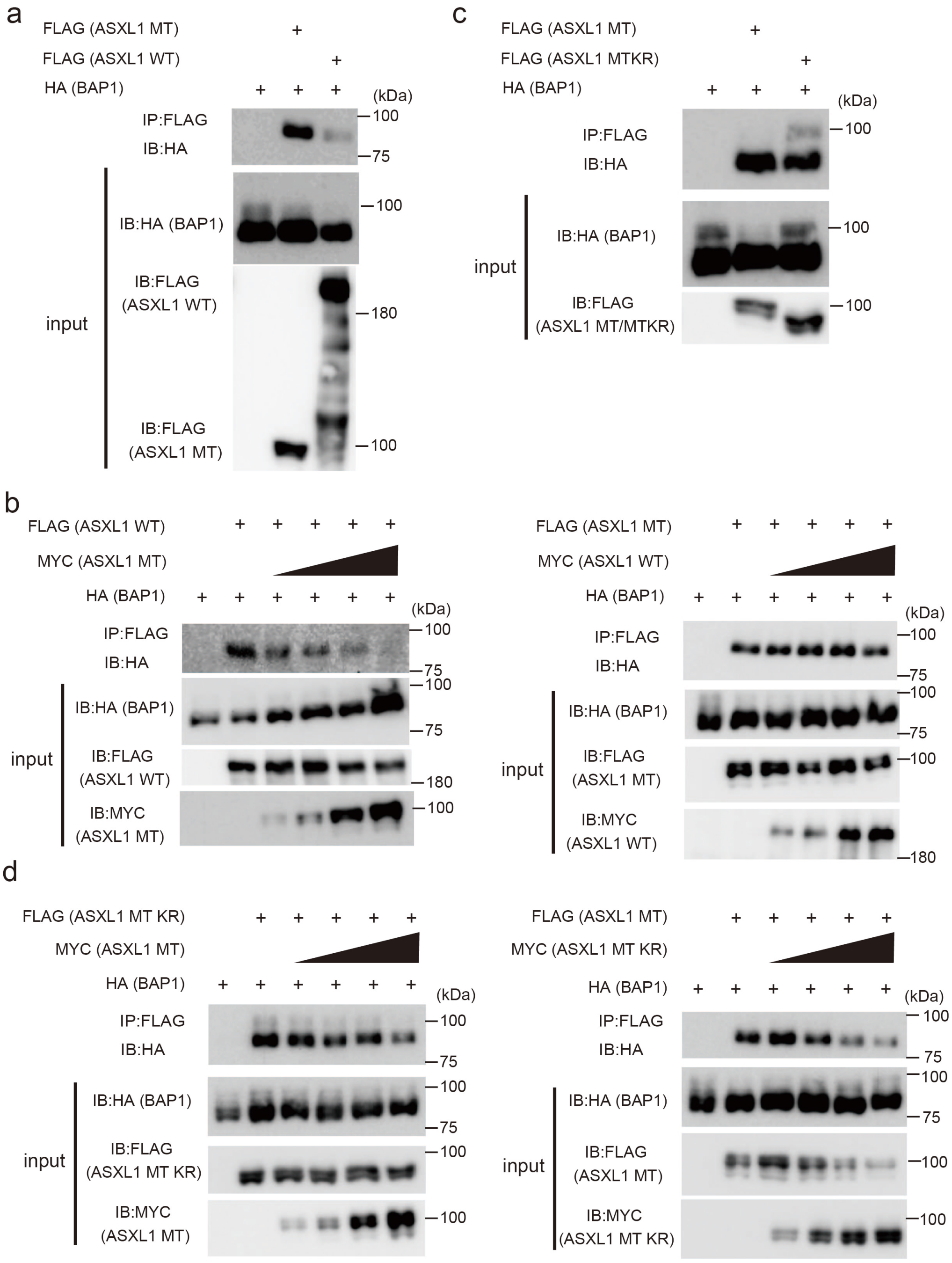


**Supplementary Figure 2. ASXL1-MT shows stronger binding affinity towards BAP1 than wild-type ASXL1. (a)** 293T cells were transfected with HA-BAP1 (1μg) together with vector, FLAG-ASXL1 MT (1μg) or FLAG-ASXL1 WT (8μg). Total cell lysates were immunoprecipitated with anti-FLAG M2 antibody, and BAP1 bound to ASXL1 was detected anti-HA. **(b)** 293T cells were transfected with HA-BAP1 together with FLAG-ASXL1 WT (8μg) and varying amounts of MYC-ASXL1 MT (0, 0.1μg, 0.2μg, 0.5μg, 1μg) (left), FLAG-ASXL1 MT (1μg) and varying amounts of MYC-ASXL1-WT (0, 1μg, 2μg, 4μg, 8μg) (right). Total cell lysates were immunoprecipitated with anti-FLAG M2 antibody, and BAP1 bound to ASXL1 was detected with anti-HA. ASXL1 WT showed weaker binding affinity towards BAP1 compared with ASXL1-MT. **(c)** 293T cells were transfected with HA-BAP1 (1μg) together with vector, FLAG-ASXL1 MT (1μg) or FLAG-ASXL1-MT K351R (1μg). Total cell lysates were immunoprecipitated with anti-FLAG M2 antibody, and BAP1 bound to ASXL1 was detected with anti-HA. **(d)** 293T cells were transfected with HA-BAP1 together with FLAG-ASXL1-MT K351R (1μg) and varying amounts of MYC-ASXL1-MT (0, 0.2μg, 0.5μg, 1μg, 2μg) (left), FLAG-ASXL1-MT (1μg) and varying amounts of MYC-ASXL1-MT K351R (0, 0.2μg, 0.5μg, 1μg, 2μg) (right). Total cell lysates were immunoprecipitated with anti-FLAG M2 antibody, and BAP1 bound to ASXL1 was detected with anti-HA. FLAG-ASXL1 MT K351R showed similar binding affinity towards BAP1 compared with ASXL1-MT.

Supplementary Figure 3

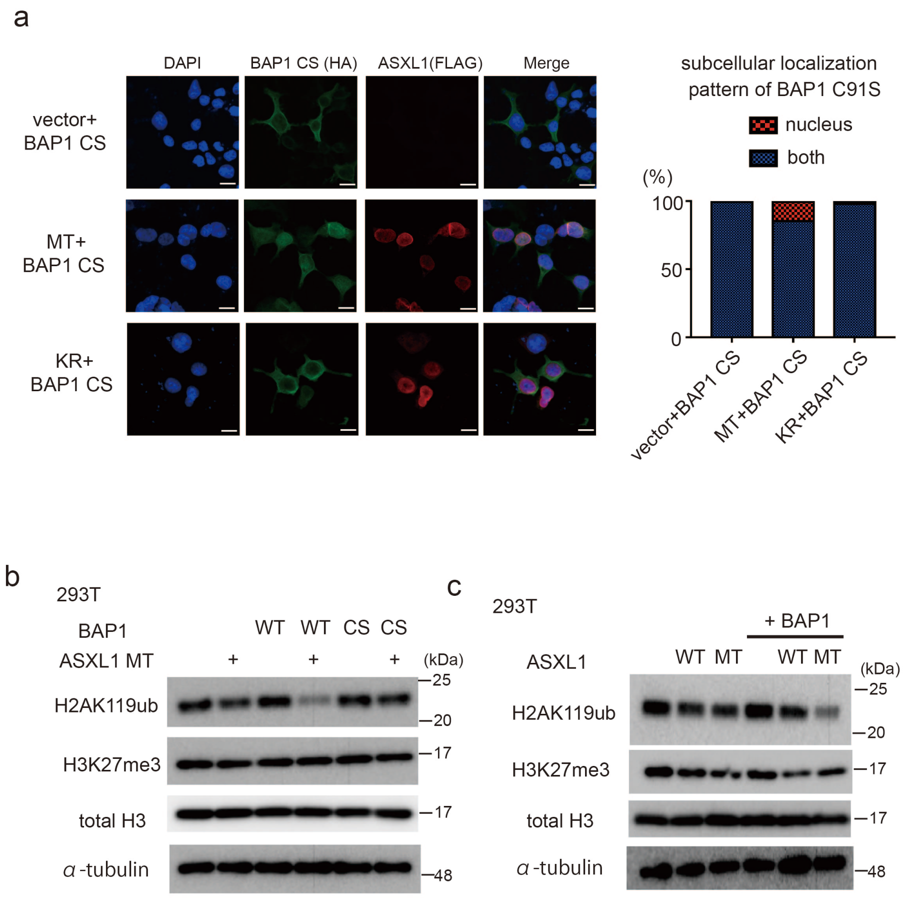


**Supplementary Figure 3. ASXL1-MT enhances nuclear retention and catalytic function of BAP1, which results in profound reduction of H2AK119ub in 293T cells. (a)** 293T cells were transfected with HA-BAP1 C91S together with vector, FLAG-ASXL1-MT (MT) or FLAG-ASXL1-MT-K351R (KR), and were stained with anti-FLAG (rabbit) or anti-HA (mouse) antibody followed by secondary anti-rabbit Alexa 568 (red) or anti-mouse Alexa 488 (green) staining. Nuclei were visualized with DAPI (Blue). Confocal laser scanning microscopy (Nikon A1) was used to observe localization of ASXL1-MT and BAP1 C91S (left). Scale bars; 10 μm. Subcellular localization of BAP1 C91S was quantified by counting 400 cells exhibiting diffuse distribution in both nucleus and cytoplasm (both) or nuclear localization (nuclear). **(b)** 293T cells were transfected with vector or ASXL1-MT (MT) together with vector, wild-type BAP1 (WT) or BAP1-C91S (CS). 48hr after transfection, cells were harvested and cell lysates extracted from them were subjected to immunoblotting with anti-H2AK119ub, anti-H3K27me3, anti-total H3, and anti-tubulin antibodies. See also Fig. 3d. **(c)** 293T cells were transfected with vector, ASXL1-MT (MT) or wild-type ASXL1 (WT) together with vector or BAP1. 48hr after transfection, cells were harvested and cell lysates extracted from them were subjected to immunoblotting with anti-H2AK119ub, anti-H3K27me3, anti-total H3, and anti-GAPDH antibodies. See also Fig. 3e.

Supplementary Figure 4

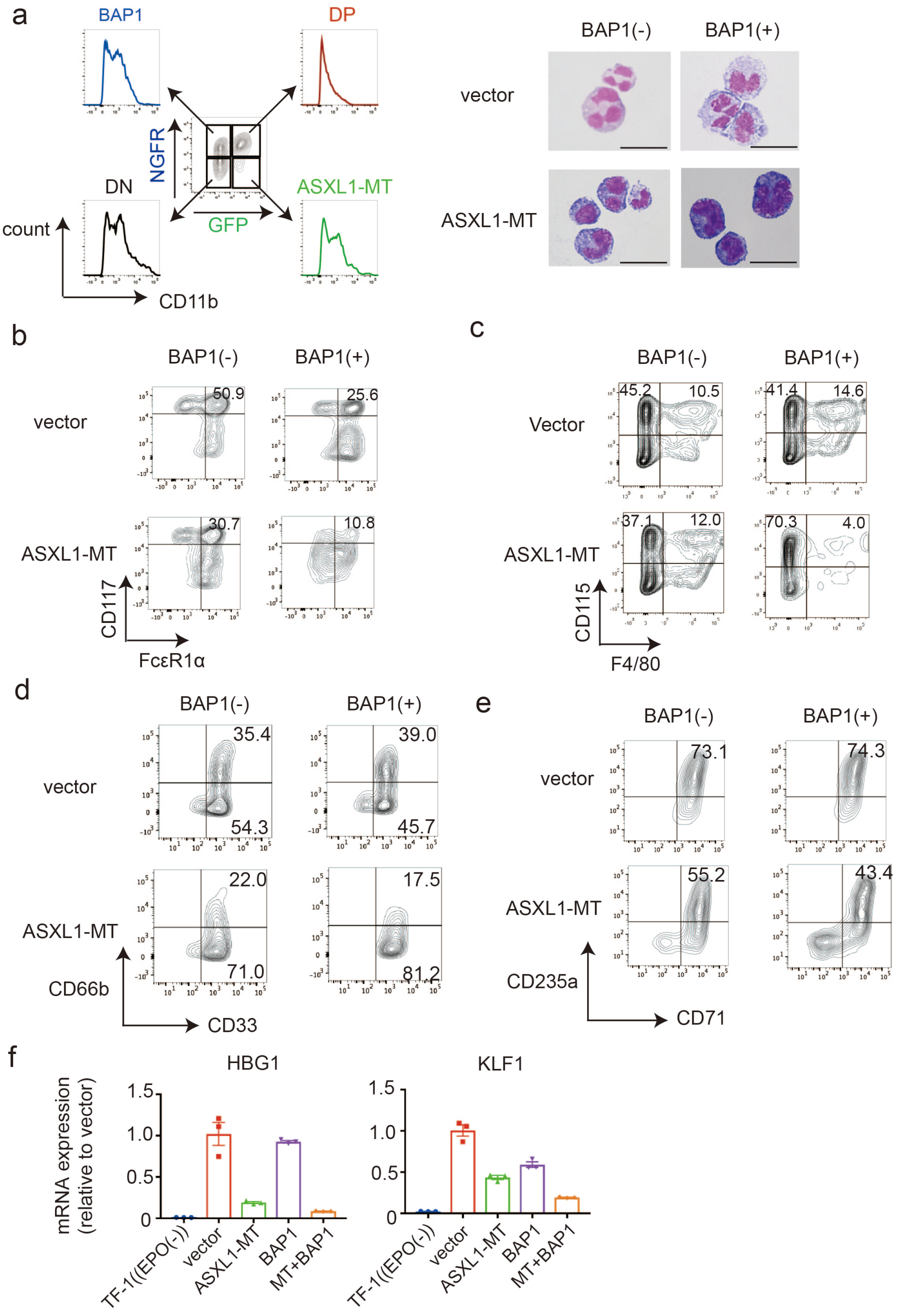


**Supplementary Figure 4. ASXL1-MT/BAP1 complex inhibits multi-lineage haematopoietic differentiation. (a)** 32Dcl3 cells were transduced with ASXL1-MT (coexpressing GFP) and BAP1 (coexpressing NGFR) (left), and were cultured with 50 ng/ml G-CSF for 6days to assess CD11b expression (left). Representative morphology of 32Dcl3 cells transduced with indicated constructs on day6 are also shown (right, scale bars; 20 μm). Coexpression of ASXL1-MT and BAP1 impaired myeloid maturation of 32Dcl3 cells more strongly than ASXL1-MT alone. **(b)** Representative FACS plots of mast cell differentiation assay on day 9. Coexpression of ASXL1-MT and BAP1 strongly impaired differentiation towards CD117$^+$ FcεIRa$^+$ mast cells. See also Fig. 4a. **(c)** Representative FACS plots of monocyte/macrophage differentiation assay on day 7. Coexpression of ASXL1-MT and BAP1 promoted differentiation towards CD115$^+$ monocytes, but failed to induce maturation towards CD115$^+$F4/80$^+$ macrophages. See also Fig. 4b.**(d)** Representative FACS plots of myeloid differentiation assay using human CB cells on day 7. Coexpression of ASXL1-MT and BAP1 impaired differentiation towards CD33$^+$CD66b$^+$ granulocytes. See also Fig. 4d. **(e)** Representative FACS plots of erythroid differentiation assay using human CB cells on day 5. Coexpression of ASXL1-MT and BAP1 impaired differentiation towards CD71$^+$CD235a$^+$ mature erythrocytes. See also Fig. 4e. **(f)** TF-1 cells were transduced with the combination of ASXL1-MT and BAP1, and were cultured in RPMI containing 10% fetal bovine serum and EPO (2 unit/ml) for 7days. RNA was extracted from the cultured cells, and expression of *KLF1* and *HBG1* were assessed by qRT-PCR. Coexpression of ASXL1-MT and BAP1 resulted in the reduced expression of *KLF1* and *HBG1*, indicating the impaired erythroid differentiation of TF-1 cells.

## Supplementary Figure 5

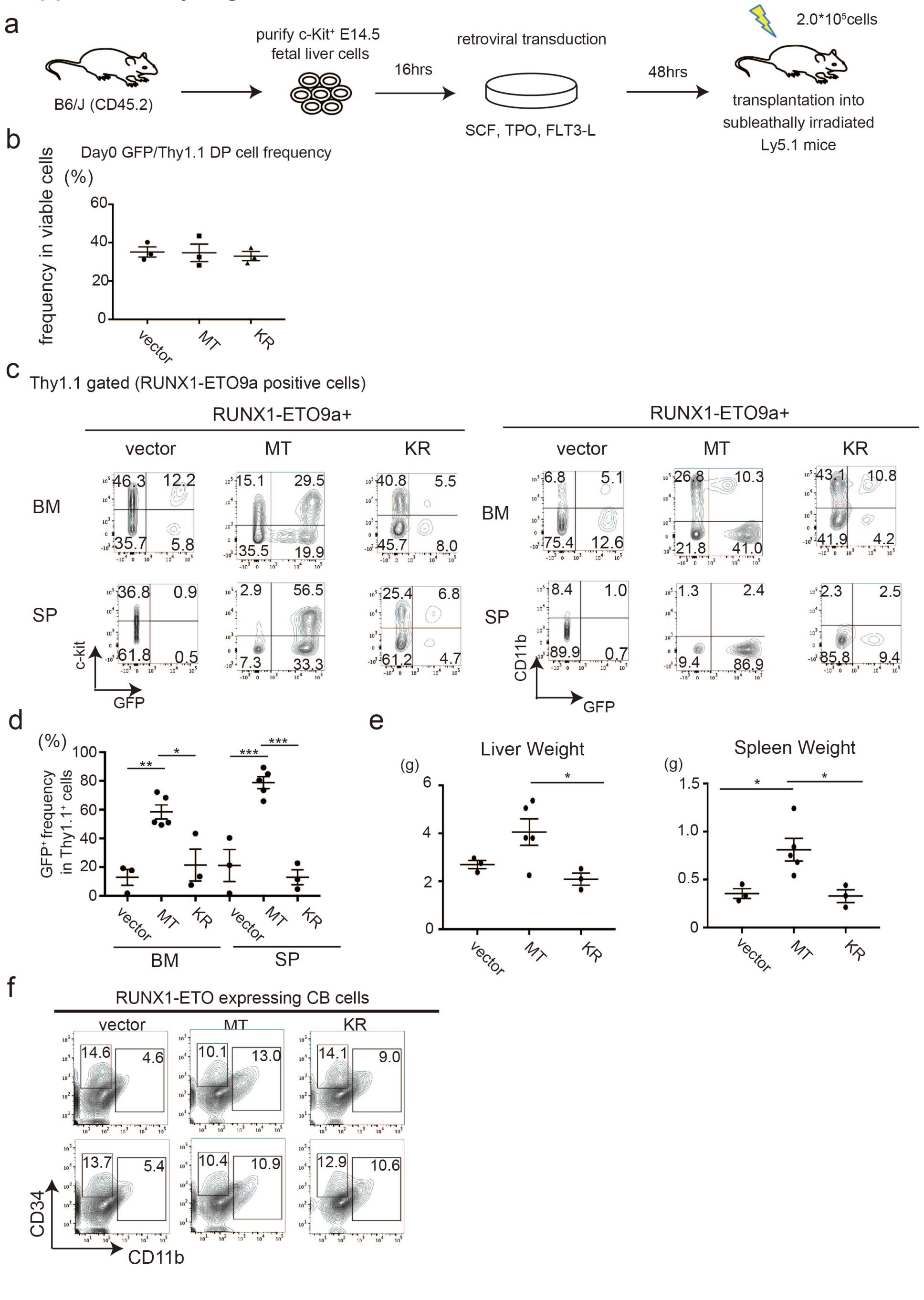


**Supplementary Figure 5. ASXL1-MT accelerates RUNX1-ETO9a-driven myeloid leukaemogenesis. (a)** Schematic presentation of experimental procedures using a mouse BMT model for RUNX1-ETO9a leukaemia. Mouse fetal liver c-kit$^+$ cells were transduced with RUNX1-ETO9a (coexpressing Thy1.1) together with vector/ASXL1-MT (MT)/ASXL1-MT-K351R (KR) (coexpressing GFP) (for experiments shown in Fig. 5c), or were transduced with RUNX1-ETO9a (coexpressing Thy1.1), vector/ASXL1-MT/ASXL1-MT-K351R (coexpressing GFP) and vector/BAP1 (coexpressing NGFR) (for experiments shown in Fig. 5d). 2×10$^5$ transduced cells were transplanted into sublethally irradiated Ly5.1 recipient mice. **(b)** Frequency of GFP/Thy1.1 double positive (DP) fraction in fetal liver cells immediately after transduction. Three independent experiments were performed, and data are shown as mean ±s.e.m. All constructs showed similar transduction efficiency. **(c)** Representative FACS plot of RUNX1-ETO9a-expressing (Thy1.1$^+$) leukaemia cells derived from bone marrow (BM) or spleen (SP) of moribund mice. There was a trend that ASXL1-MT (MT) -expressing cells contained more c-kit+ cells and less CD11b+ cells compared with vector- or ASXL1-MT-K351R (KR)-expressing cells, indicating the immature phenotype of ASXL1-MT-expressing RUNX1-ETO9a leukaemia cells. **(d)** Frequency of vector/ASXL1-MT (MT) /ASXL1-MT-K351R (KR)-expressing (GFP$^+$) cells in RUNX1-ETO9a-expressing (Thy1.1$^+$) cells derived from BM or SP of moribund mice. Leukaemia cells expressing both RUNX1-ETO9a and ASXL1-MT were enriched in both BM and SP. Data are shown as mean ± s.e.m. (vector, KR n=3, MT n=5). *P<0.05, **P<0.01, ***P<0.001, one-way ANOVA with Tukey's multiple-comparisons test. **(e)** The weights of liver and spleen taken form moribund mice were assessed. Data are shown as mean ± s.e.m. (vector, KR n=3, MT n=5). Mice transplanted with cells expressing RUNX1-ETO9a and ASXL1-MT displayed hepatosplenomegaly compared to other groups, indicating the more aggressive organ infiltration of RUNX1-ETO leukaemia cells expressing ASXL1-MT. *P<0.05, one-way ANOVA with Tukey's multiple-comparisons test. **(f)** RUNX1-ETO transduced human CB CD34$^+$ cells were transduced with vector/MT/KR (co-expressing GFP). Expression of CD34 and CD11b on day11. Shown are FACS plots of two independent experiments.

## Supplementary Figure 6

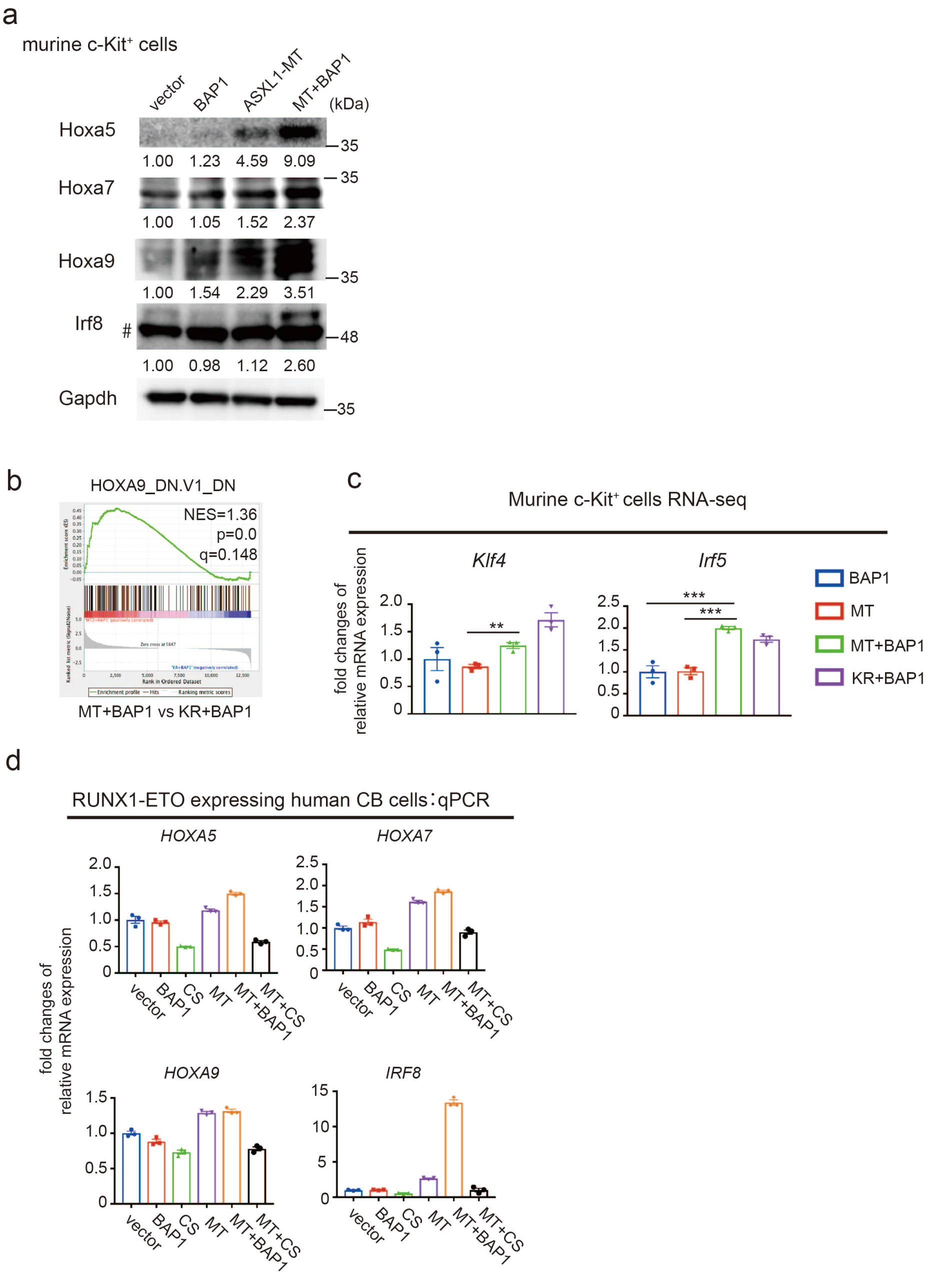


**Supplementary Figure 6. *Hox*a genes and *Irf8* are downstream targets of ASXL1-MT/BAP1 complex. (a)** Murine bone marrow c-Kit+ cells were transduced with ASXL1-MT (MT) or ASXL1-MT-K351R (KR) (coexpressing blasticidin resistant gene) together with vector or BAP1 (coexpressing puromycin resistant gene), and were cultured in M3234 containing 20 ng/ml SCF, 10 ng/ml IL-3 and 10 ng/ml IL-6. After the selection with blasticidin and puromycin for three days, total cell lysates were subjected to immunoblotting for indicated antibodies. Cells expressing ASXL1-MT/BAP1 increased expression of Hoxa5, Hoxa7, Hoxa9 and Irf8 protein**.** (#): non-specific band. **(b)** Gene set enrichment analysis (GSEA) revealed that *Hoxa9* target genes were highly expressed in mouse haematopoietic stem/progenitor cells (HSPCs) expressing ASXL1-MT (MT) and BAP1 compared with those in cells expressing ASXL1-MT-K351R (KR) and BAP1. **(c)** Relative expression of *Klf4* and *Irf5,* known target genes of *Irf8*, in HSPCs transduced with indicated constructs. Expression of each gene in cells expressing BAP1 alone was set as 1. n=3 for each group, and data are shown as mean ± s.e.m, $^{**}P<0.01$, $^{***}P<0.001$, one-way ANOVA with Tukey's multiple-comparisons test. **(d)** RUNX1-ETO-expressing CB cells were transduced with vector or ASXL1-MT (MT) (coexpressing GFP) in combination with vector, BAP1 or BAP1-C91S (CS) (coexpressing NGFR). GFP/NGFR double positive cells were sorted by FACSAria 48hr after transduction. Relative mRNA levels of *HOXA5, HOXA7, HOXA9* and *IRF8* were analyzed by qRT-PCR. Results were normalized to *GAPDH*, with the relative mRNA level in vector-transduced cells set at 1. Data are shown as mean ± s.e.m. of triplicate wells.

## Supplementary Figure 7

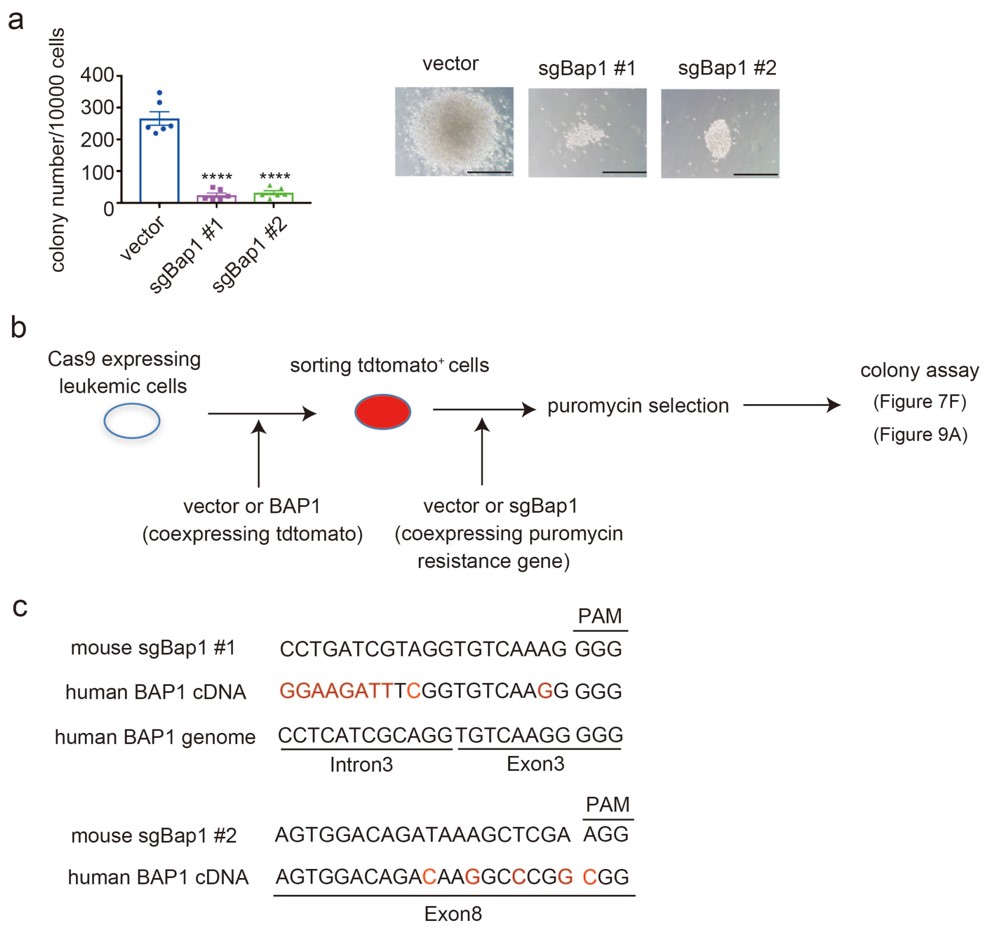


**Supplementary Figure 7. Bap1 depletion inhibits the colonogenicity of myeloid leukaemia cells, which was reversed by expression of human BAP1. (a)** An independent experiment to assess the effect of Bap1 depletion in cSAM cells. cSAM cells were transduced with Cas9 and Bap1-targeting sgRNAs, and were cultured in methylcellulose medium. Colony numbers were counted on day 7. Six independent experiments were performed, and data are shown as mean ± s.e.m. ****P<0.0001, one-way ANOVA with Tukey's multiple-comparisons test (left). Representative photos of colonies on day 7 are also shown (right). Scale bars; 200μm. See also Fig. 7f. **(b)** Schematic presentation of experimental procedures for experiments shown in Fig. 7f and 9a. cSAM cells or MLL-AF9 cells (leukaemia cells generated from mouse bone marrow c-Kit$^+$ cells) were transduced with Cas9 together with vector or human BAP1 (coexpressing tdtomato). The tdTomato$^+$ cells were sorted by FACSAria 48 hrs after transduction, and were then transduced with vector or sgRNAs (coexpressing puromycin resistant gene) targeting mouse Bap1. After the selection with puromycin, cells were cultured in methylcellulose medium. **(c)** Sequences of human BAP1 cDNA and two sgRNAs targeting murine BAP1 (sgBap1 #1 and sgBap1 #2). Neither sgBap1 #1 nor sgBap1 #2 cuts human BAP1 cDNA by the CRISPR/Cas9 system. PAM; PAM sequence.

## Supplementary Figure 8

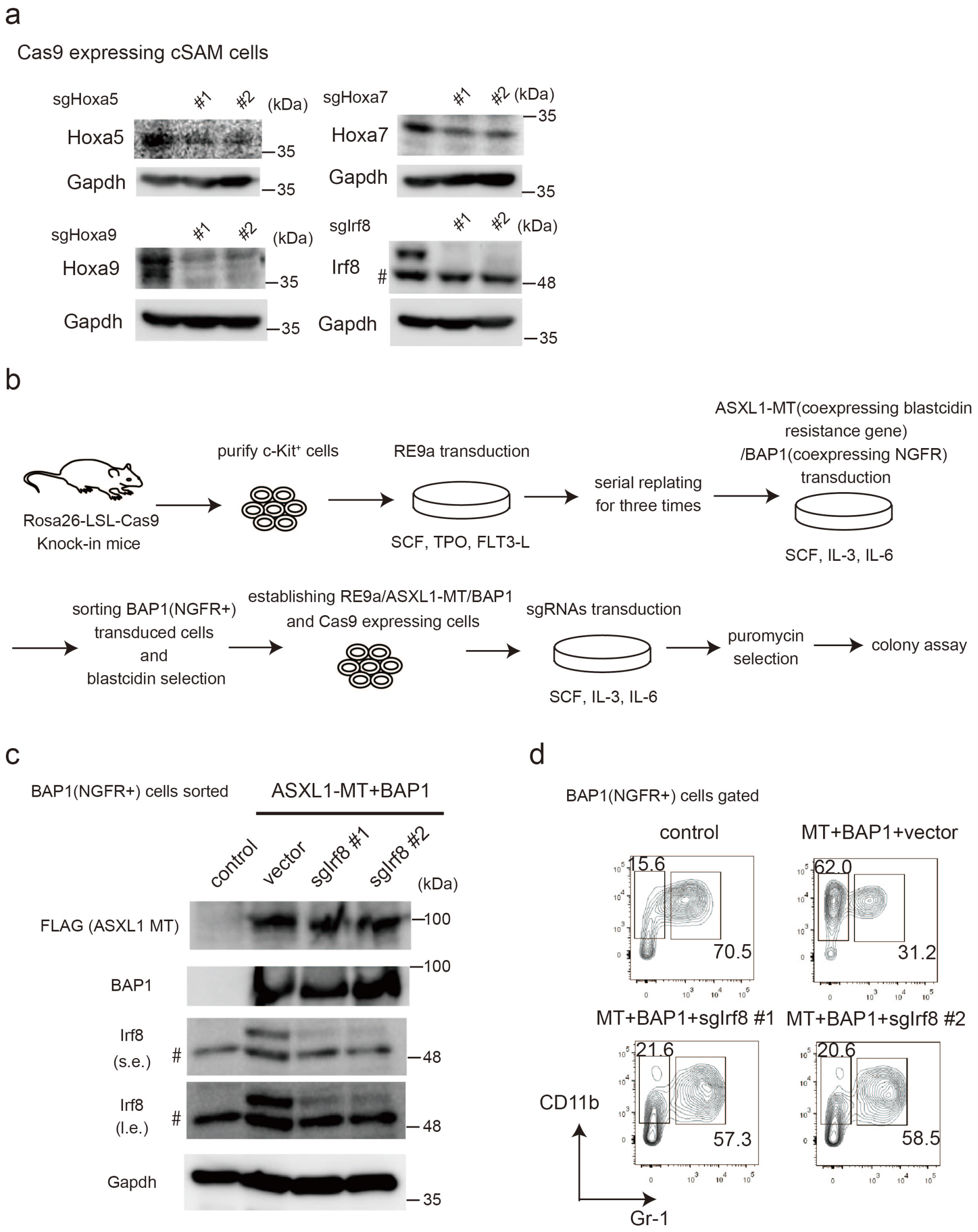


**Supplementary Figure 8. Depletion of Hoxa genes and Irf8 using CRISPR/Cas9 in cSAM cells and murine bone marrow cells. (a)** Cas9 expressing cSAM cells were transduced with control vector or two independent sgRNAs targeting Hoxa5, Hoxa7, Hoxa9 or Irf8. Cell lysates were extracted from these cells and were subjected to immunoblotting. Hoxa5**,** Hoxa7**,** Hoxa9 and Irf8 were efficiently depleted by the corresponding sgRNAs. (#): non-specific band. **(b)** Schematic presentation of experimental procedures for experiments shown in Figure 8b. **(c)** Cell lysates extracted from NGFR positive (BAP1 transduced) cells in Fig 8f were subjected to immunoblotting for indicated antibodies. Cells expressing ASXL1-MT/BAP1 expressed high-level of Irf8 protein, which was downregulated by the two sgRNAs targeting Irf8. (#): non-specific band. **(d)** Representative FACS plots of granulocyte (Gr-1$^{+}$CD11b$^{+}$) and monocyte (Gr-1$^{-}$CD11b$^{+}$) differentiation assays performed in Fig 8f.

## Supplementary Figure 9

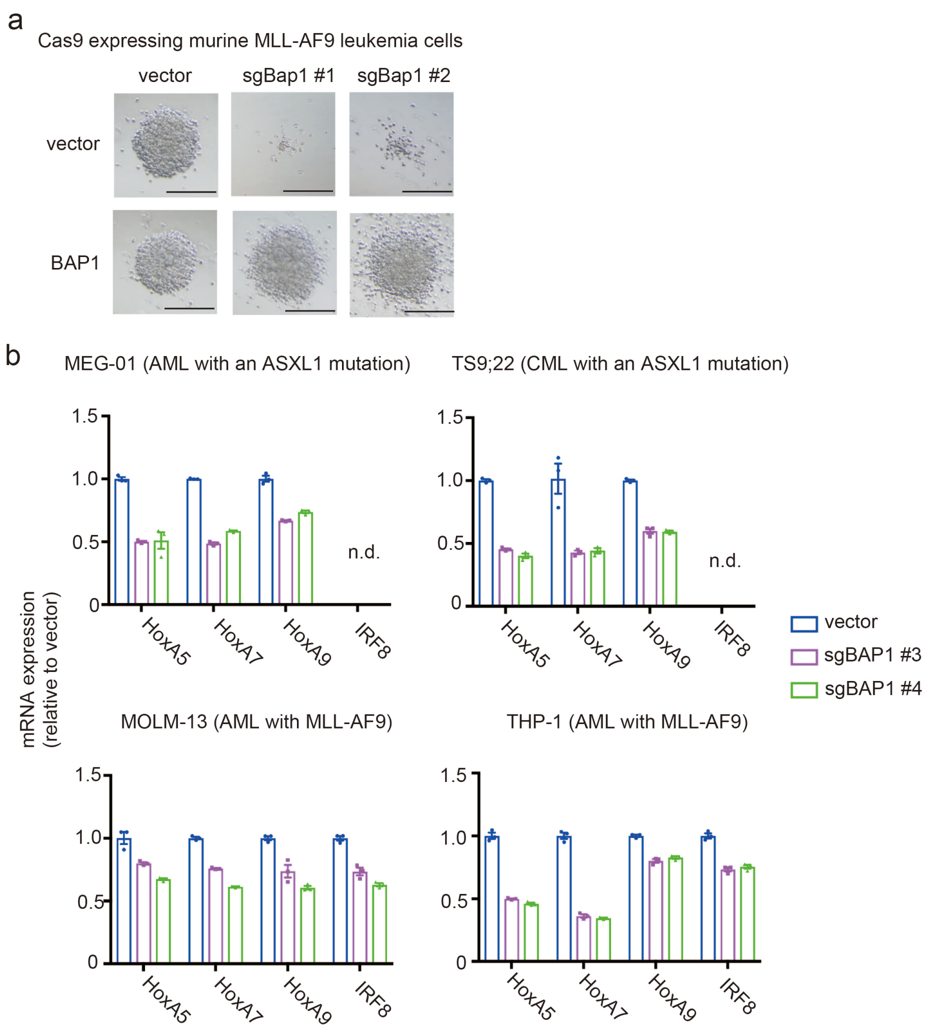


**Supplementary Figure 9. BAP1 depletion results in the reduced expression of *HOXA* genes in human leukaemia cells. (a)** Murine bone marrow c-Kit$^{+}$ cells were transformed by MLL-AF9. The MLL-AF9 leukaemia cells were transduced with Cas9. Vector or human BAP1-transduced (tdTomato$^{+}$) cells were sorted, and were then transduced with vector or two independent sgRNAs targeting mouse Bap1 (sgBap1 #1, #2). Cells were cultured in semisolid medium. Representative photos of colonies derived from indicated cells on day 7 are shown. Scale bars; 200μm. See also Fig. 9a. **(b)** Human leukaemia cell lines MEG-01, TS9;22 (harboring an ASXL1 mutation), MOLM-13 and THP-1 (harboring MLL-AF9) were transduced with Cas9 (coexpressing blasticidin resistant gene) together with a vector or two gRNAs targeting human BAP1 (sgBAP1 #3/sgBAP1 #4) constructs (coexpressing puromycin resistant gene). After the selection with blasticidin and puromycin for three days, expression of *HOXA5, HOXA7, HOXA9* and *IRF8* in these cells were assessed by qRT-PCR. Results were normalized to *GAPDH*, with the relative mRNA level in vector-transduced cells set at 1. Data are shown as mean ± s.e.m. of triplicate wells. BAP1 depletion resulted in the reduced expression of *HOXA* genes and *IRF8* in MEG-01, TS9;22, MOLM-13 and THP-1 cells. n.d.; not detected.

## Supplementary Figure 10

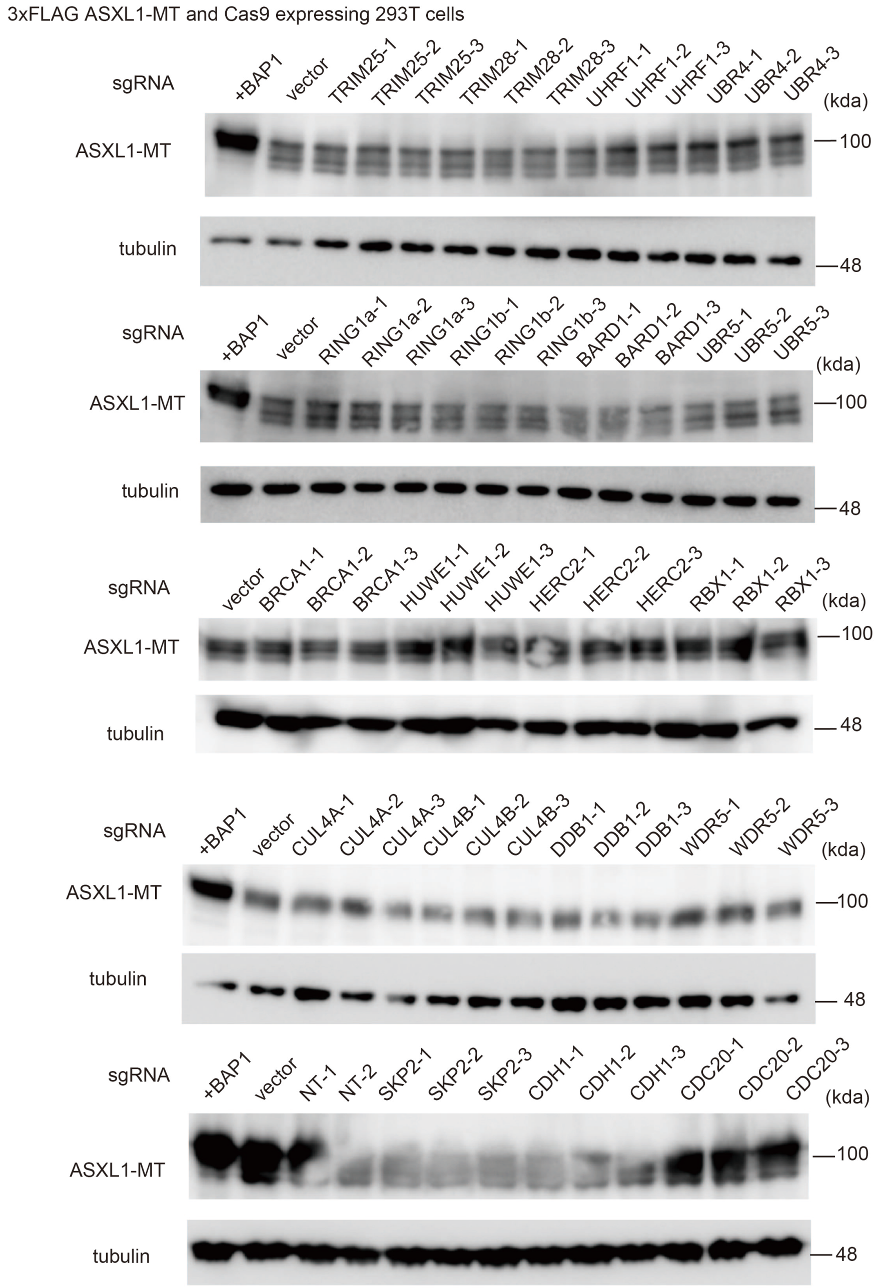


**Supplementary Figure 10. Depletion of ubiquitination-related proteins does not inhibit monoubiquitination of ASXL1-MT.**
293T cells that stably express 3xFLAG tagged ASXL1-MT and Cas9 were transduced with indicated sgRNAs. Cell lysates extracted from them were subjected to immunoblotting with anti-FLAG and anti-tubulin antibodies. None of these sgRNAs inhibited monoubiquitination of ASXL1-MT. The list of sgRNA sequences is provided in Supplementary Table 1.

## Supplementary Figure 11

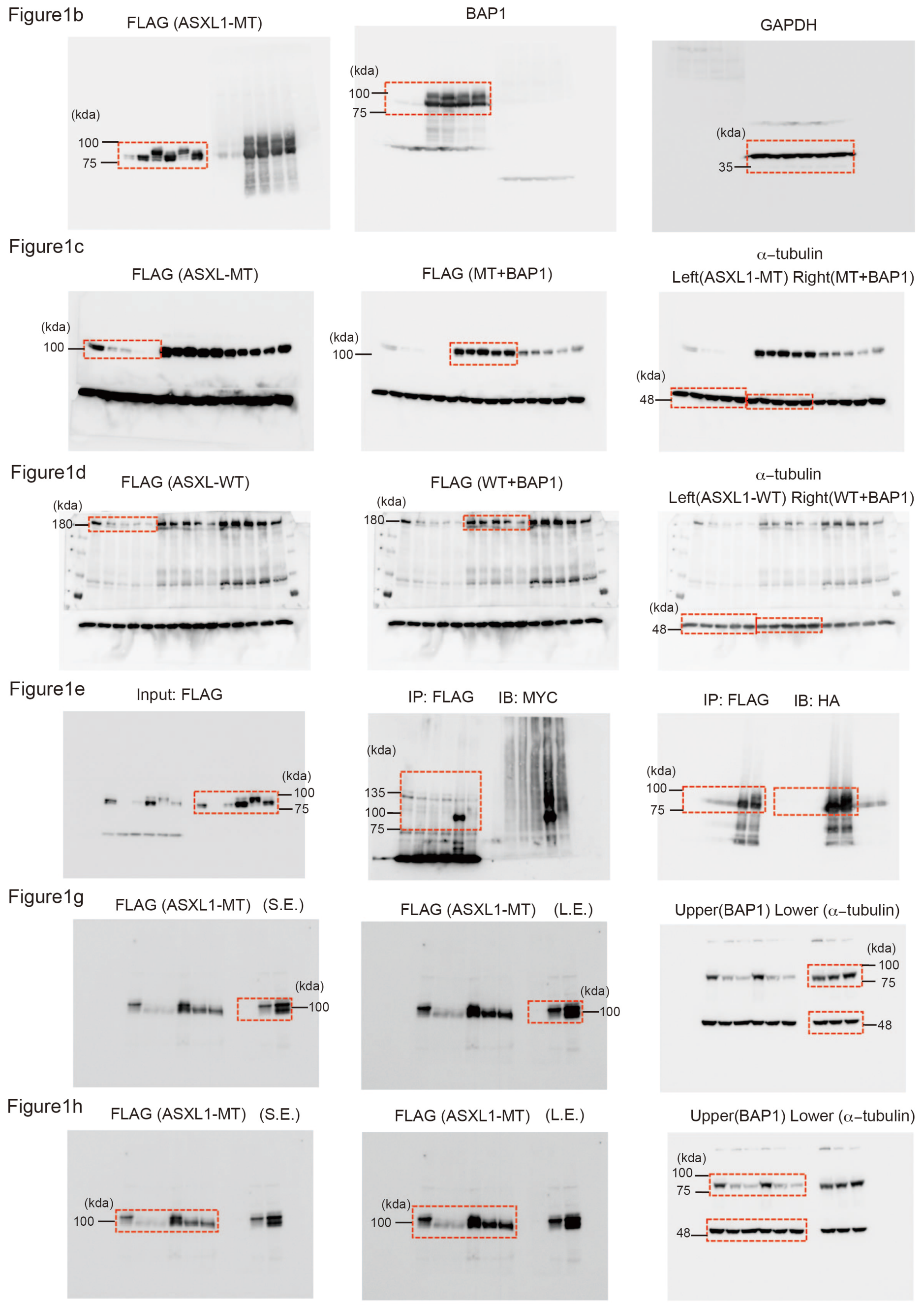


**Supplementary Figure 11. Uncropped immunoblotting images from Figure 1. The red boxes indicate the cropped images.**

# Supplementary Figure 12

Figure2c

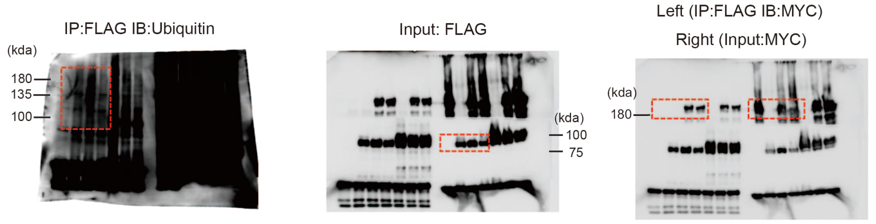


Figure2d

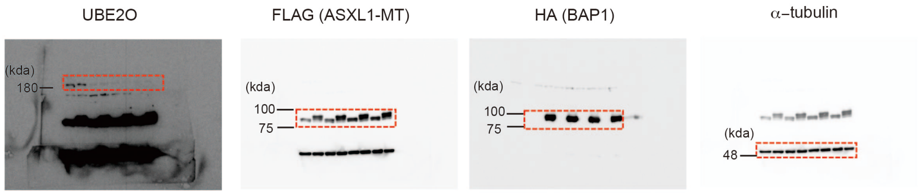


Figure2e

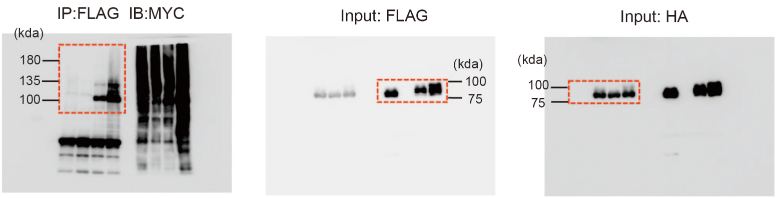


**Supplementary Figure 12. Uncropped immunoblotting images from Figure 2. The red boxes indicate the cropped images.**

# Supplementary Figure 13

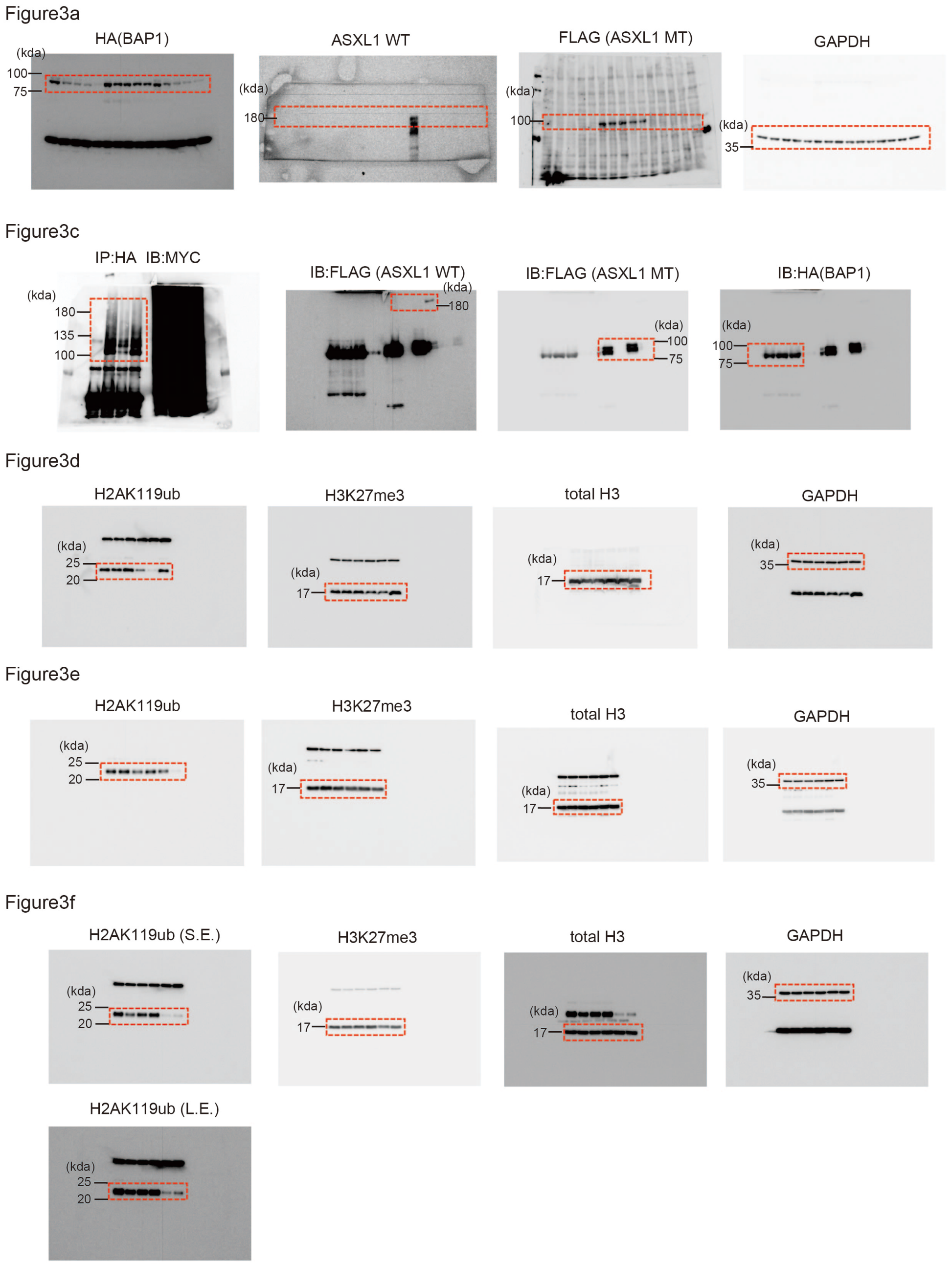


**Supplementary Figure 13. Uncropped immunoblotting images from Figure 3. The red boxes indicate the cropped images.**

# Supplementary Figure 14

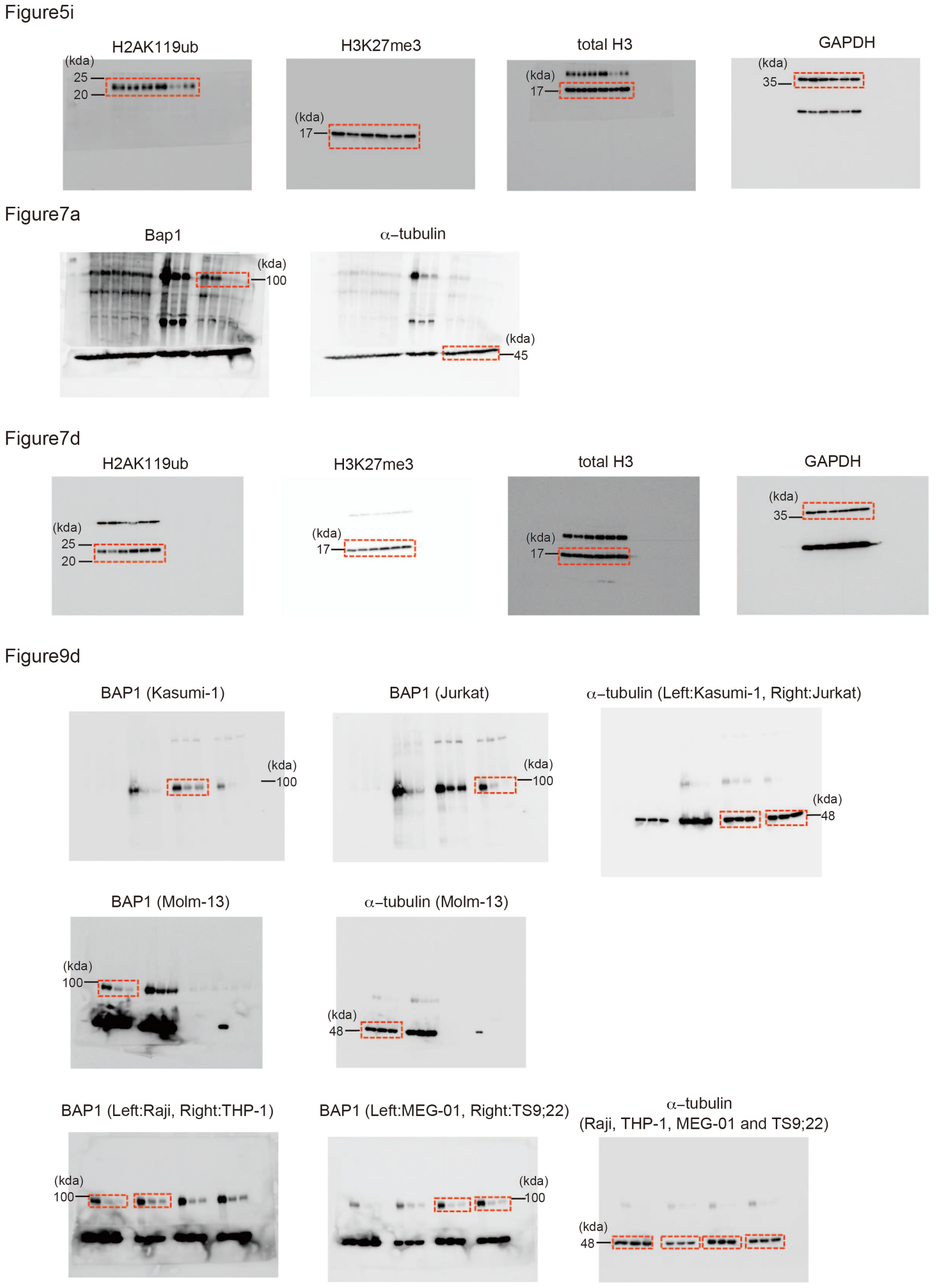


**Supplementary Figure 14. Uncropped immunoblotting images from Figure 5i, 7a, 7d and 9d. The red boxes indicate the cropped images.**

| sgRNA | sequence | sgRNA | sequence |
|---|---|---|---|
| TRIM25-1 | TGTTCCGGGGCTCCAAACGT | HERC2-1 | AGCAGCTACGATAAGAACCA |
| TRIM25-2 | CATGTACAGTCAGATCAACG | HERC2-2 | GATGTGGTCAAAGTCCGCTG |
| TRIM25-3 | CCTCAGAGACCACCTCGACA | HERC2-3 | GATCGTGACACCCAACGGGA |
| TRIM28-1 | TGAAACTTCATCTCGCCATG | RBX-1 | ATGGATGTGGATACCCCGAG |
| TRIM28-2 | CTTCCCAGGCAGTACCACTG | RBX-2 | CAGAAGAGTGTACTGTCGCA |
| TRIM28-3 | CCAGCGGGTGAAGTACACCA | RBX-3 | GTTATCAACCACAATATCCC |
| UHRF-1 | TCAGACAAGTCCTCCACCCA | CUL4A-1 | AGTTCTGCAGCACATAGGTG |
| UHRF-2 | GCGGGAACTCTACGCCAACG | CUL4A-2 | TATGCTCACAGCAAAGCATG |
| UHRF-3 | TGCTCGGGACACGAACATGG | CUL4A-3 | GGAAGCAGACCTCGATCACG |
| UBR4-1 | GGTGTCAATGAGTTGCAGCG | CUL4B-1 | CTCTTCAGTAGAGTTCGAGG |
| UBR4-2 | GAAGATCTCGATATATGACG | CUL4B-2 | GCTTCTTCTGTATCGGTACG |
| UBR4-3 | GGAACCGATTGATATAGCGT | CUL4B-3 | AGCATGTGGTACTTACTGGG |
| RING1A-1 | ACTGCATTGTCACAGCCCTA | DDB1-1 | CATTGTCGATATGTGCGTGG |
| RING1A-2 | GAAGAATACGATGACCACCA | DDB1-2 | GGATAGCCATCTGAATTGAG |
| RING1A-3 | TAGGACACTCCTTGTTCCTG | DDB1-3 | CATTAGGGTCCACTCGATTG |
| RING1B-1 | ACAAAGAATGTCCTACCTGT | WDR5-1 | TTCAGTTTGACGAAAGCGTG |
| RING1B-2 | TAAACACTCCTTTGTAGTCA | WDR5-2 | GGCACGCACCTTGCTCTGAG |
| RING1B-3 | ATCATCACAGCCCTTAGAAG | WDR5-3 | AGGGAATATCCGATGTAGCC |
| BARD1-1 | GCTGTGAAAAGAAATCATAG | NT-1 | ACGGAGGCTAAGCGTCGCAA |
| BARD1-2 | ATGTCCAGTGTGTTACACCC | NT-2 | CGCTTCCGCGGCCCGTTCAA |
| BARD1-3 | ACTTTACGTTTGCATGAAGG | SKP2-1 | GTTGGTCCATAAATGATCGT |
| UBR5-1 | TTGTTGTCTAAGAACGACGA | SKP2-2 | AACACTGAGACAGTATGCCG |
| UBR5-2 | TTGATCCTTCAAACTTACGC | SKP2-3 | ACCGACTGAGTGATAGGTGT |
| UBR5-3 | TTTCTCCCAACAATCACAGT | CDH1-1 | CCTCACCGACGCGTGCCGTG |
| BRCA1-1 | AGAAACCTACAACTCATGGA | CDH1-2 | GGCACTCCACAGGTACACGC |
| BRCA1-2 | AAATATGTGGTCACACTTTG | CDH1-3 | CCACTGCAGTGTATCGACAC |
| BRCA1-3 | AAATCTTAGAGTGTCCCATC | CDC20-1 | ATGATAACTTGGTCAATGTG |
| HUWE1-1 | GCTGAACTTCACCTGAATCG | CDC20-2 | TCCCAGAACTCCAATCCACA |
| HUWE1-2 | CACCCTCCTCCAATCATGGG | CDC20-3 | AAAACAGCGCCATAGCCTCA |
| HUWE1-3 | AATCTTAACAACTTACCAAG | | |

**Supplementary Table 1: The list of sgRNA sequences used in Supplementary Figure 10.**
