# Supplementary material for: Mutant ASXL1 cooperates with BAP1 to promote myeloid leukaemogenesis
Source: Nat Commun. 2018 Jul 16;9:2733. doi: 10.1038/s41467-018-05085-9 (PMC6048047; doi:10.1038/s41467-018-05085-9)
Supplement: Supplementary file 3 — Description of Additional Supplementary Files [file 41467_2018_5085_MOESM3_ESM.pdf]

Description of Additional Supplementary Files

File Name: Supplementary Data 1
Description: The list of posttranslational modifycations (PTMs) of ASXL1-MT in the absence of BAP1 coexpression. 293T cells were transfected with ASXL1-MT alone, and the PTMs of ASXL1-MT were analyzed with Nano-LC MS/MS.

File Name: Supplementary Data 2
Description: The list of posttranslational modifycations (PTMs) of ASXL1-MT in the presence of BAP1 coexpression. 293T cells were transfected with ASXL1-MT and BAP1, and the PTMs of ASXL1-MT were analyzed with Nano-LC MS/MS. Note that the ubiquitin-derived diglycine on K351 of ASXL1-MT (colored by yellow) was detected only when BAP1 was coexpressed in cells.

File Name: Supplementary Data 3
Description: The list of proteins that interact with ASXL1-MT in the absence of BAP1 coexpression. 293T cells were transfected with ASXL1-MT alone, and 622 ASXL1-MT-binding proteins were identified by nano LC-MS/MS. See also Fig. 2, a and b.

File Name: Supplementary Data 4
Description: The list of proteins that interact with ASXL1-MT in the presence of BAP1 coexpression. 293T cells were transfected with ASXL1-MT and BAP1, and 427 ASXL1-MT-binding proteins were identified by nano LC-MS/MS. See also Fig. 2, a and b.

File Name: Supplementary Data 5
Description: The RNA-seq data of murine c-kit+ cells transduced with ASXL1-MT (MT) or ASXL1-MT-K351R (KR) together with vector or BAP1.
